# 8-Hydr­oxy-2-methyl­quinolinium dichlorido(2-methyl­quinolin-8-olato-κ^2^ *N*,*O*)zincate(II) methanol solvate

**DOI:** 10.1107/S1600536809014202

**Published:** 2009-04-22

**Authors:** Elham Sattarzadeh, Gholamhossein Mohammadnezhad, Mostafa M. Amini, Seik Weng Ng

**Affiliations:** a Department of Chemistry, General Campus, Shahid Beheshti University, Tehran 1983963113, Iran; b Department of Chemistry, University of Malaya, 50603 Kuala Lumpur, Malaysia

## Abstract

The reaction of zinc chloride and 2-methyl-8-hydroxy­quinoline in methanol yielded the title monosolvated salt, (C_10_H_10_NO)[ZnCl_2_(C_10_H_8_NO)]·CH_3_OH, which has the Zn atom within a distorted Cl_2_NO tetra­hedral coordination geometry. Supra­molecular chains feature in the crystal structure, comprising all components of the structure stabilized by a combination of O—H⋯O, N—H⋯O and O—H⋯Cl hydrogen bonding.

## Related literature

Unlike 8-hydroxy­quinoline, which yields a large number of metal derivatives, 2-methyl-8-hydroxy­quinoline forms only a small number of metal chelates. Besides a related acetate salt (Sattarzadeh *et al.*, 2009 ▶), there is only one crystal structure report of another zinc derivative; for aqua­bis(2-methyl­quinolin-8-ato)zinc, see: da Silva *et al.* (2007 ▶).
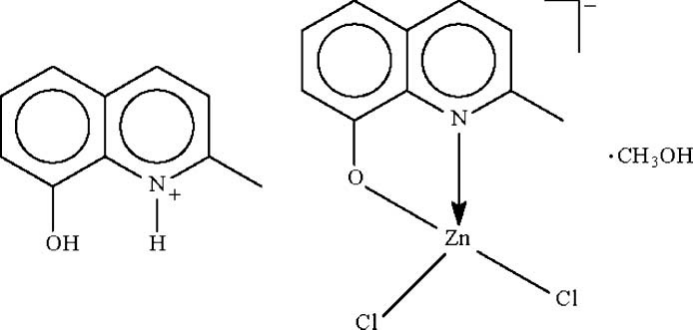


## Experimental

### 

#### Crystal data

(C_10_H_10_NO)[ZnCl_2_(C_10_H_8_NO)]·CH_4_O
*M* *_r_* = 486.68
Monoclinic, 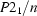

*a* = 10.0717 (2) Å
*b* = 13.7886 (3) Å
*c* = 15.4828 (3) Å
β = 105.48 (1)°
*V* = 2072.15 (7) Å^3^
*Z* = 4
Mo *K*α radiation
μ = 1.47 mm^−1^
*T* = 100 K
0.32 × 0.12 × 0.08 mm

#### Data collection

Bruker SMART APEX diffractometer
Absorption correction: multi-scan (*SADABS*; Sheldrick, 1996 ▶) *T*_min_ = 0.651, *T*_max_ = 0.892
18982 measured reflections
4753 independent reflections
3600 reflections with *I* > 2σ(*I*)
*R*_int_ = 0.036

#### Refinement

*R*[*F* ^2^ > 2σ(*F* ^2^)] = 0.039
*wR*(*F* ^2^) = 0.108
*S* = 1.02
4753 reflections
277 parameters
3 restraints
H atoms treated by a mixture of independent and constrained refinement
Δρ_max_ = 1.08 e Å^−3^
Δρ_min_ = −1.00 e Å^−3^

### 

Data collection: *APEX2* (Bruker, 2008 ▶); cell refinement: *SAINT* (Bruker, 2008 ▶); data reduction: *SAINT*; program(s) used to solve structure: *SHELXS97* (Sheldrick, 2008 ▶); program(s) used to refine structure: *SHELXL97* (Sheldrick, 2008 ▶); molecular graphics: *X-SEED* (Barbour, 2001 ▶); software used to prepare material for publication: *publCIF* (Westrip, 2009 ▶).

## Supplementary Material

Crystal structure: contains datablocks global, I. DOI: 10.1107/S1600536809014202/tk2423sup1.cif

Structure factors: contains datablocks I. DOI: 10.1107/S1600536809014202/tk2423Isup2.hkl

Additional supplementary materials:  crystallographic information; 3D view; checkCIF report

## Figures and Tables

**Table 1:** Hydrogen-bond geometry (Å, °)

*D*—H⋯*A*	*D*—H	H⋯*A*	*D*⋯*A*	*D*—H⋯*A*
O2—H2O⋯O1	0.84 (1)	1.70 (1)	2.534 (3)	177 (4)
O3—H3O⋯Cl1^i^	0.84 (1)	2.47 (3)	3.239 (4)	153 (5)
N2—H2N⋯O3	0.88 (1)	1.87 (2)	2.727 (4)	163 (3)

Symmetry code: (i) 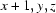.

**Fig. 1.**
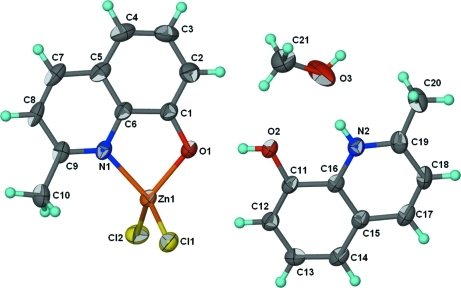
Thermal ellipsoid plot (Barbour, 2001) of [C10H10NO][Zn(C10H8NO)Cl2].CH3OH; ellipsoids are drawn at the 70% probability level and H atoms of arbitrary radius.

## supplementary crystallographic information

### Experimental

Zinc chloride (0.10 g, 0.75 mmol) and 2-methyl-8-hydroxyquinoline (0.24 g, 1.5 mmol) were loaded into a convection tube; the tube was filled with dry methanol and kept at 333 K. Crystals were collected from the side arm after several days.

### Refinement

Carbon-bound H-atoms were placed in calculated positions (C—H 0.95 to 0.98 Å) and were included in the refinement in the riding model approximation, with *U*(H) set to 1.2 to 1.5*U*(C). The O–H and N–H hydrogen atoms were located in a difference Fourier map, and were refined with distance restraints of O–H 0.84±0.01 Å and N–H 0.88±01 Å; their temperature factors were freely refined.

The final difference Fourier map had a large peak/deep hole in the vicinity of the O3 atom.

### Crystal data

**Table:**

(C_10_H_10_NO)[ZnCl_2_(C_10_H_8_NO)]·CH_4_O	*F*(000) = 1000
*M**_r_* = 486.68	*D*_x_ = 1.560 Mg m^−^^3^
Monoclinic, *P*2_1_/*n*	Mo *K*α radiation, λ = 0.71073 Å
Hall symbol: -P 2yn	Cell parameters from 4908 reflections
*a* = 10.0717 (2) Å	θ = 2.6–27.1°
*b* = 13.7886 (3) Å	µ = 1.47 mm^−^^1^
*c* = 15.4828 (3) Å	*T* = 100 K
β = 105.48 (1)°	Block, yellow
*V* = 2072.15 (7) Å^3^	0.32 × 0.12 × 0.08 mm
*Z* = 4	

### Data collection

**Table:**

Bruker SMART APEX diffractometer	4753 independent reflections
Radiation source: fine-focus sealed tube	3600 reflections with *I* > 2σ(*I*)
graphite	*R*_int_ = 0.036
ω scans	θ_max_ = 27.5°, θ_min_ = 2.0°
Absorption correction: multi-scan (*SADABS*; Sheldrick, 1996)	*h* = −13→13
*T*_min_ = 0.651, *T*_max_ = 0.892	*k* = −17→17
18982 measured reflections	*l* = −20→20

### Refinement

**Table:**

Refinement on *F*^2^	Primary atom site location: structure-invariant direct methods
Least-squares matrix: full	Secondary atom site location: difference Fourier map
*R*[*F*^2^ > 2σ(*F*^2^)] = 0.039	Hydrogen site location: inferred from neighbouring sites
*wR*(*F*^2^) = 0.108	H atoms treated by a mixture of independent and constrained refinement
*S* = 1.02	*w* = 1/[σ^2^(*F*_o_^2^) + (0.0493*P*)^2^ + 2.8684*P*] where *P* = (*F*_o_^2^ + 2*F*_c_^2^)/3
4753 reflections	(Δ/σ)_max_ = 0.001
277 parameters	Δρ_max_ = 1.08 e Å^−^^3^
3 restraints	Δρ_min_ = −1.00 e Å^−^^3^

### Fractional atomic coordinates and isotropic or equivalent isotropic displacement parameters (Å^2^)

**Table:**

	*x*	*y*	*z*	*U*_iso_*/*U*_eq_	
Zn1	0.50382 (3)	0.63261 (3)	0.23689 (2)	0.02655 (11)	
Cl1	0.48941 (8)	0.76378 (6)	0.31836 (5)	0.03149 (18)	
Cl2	0.39683 (8)	0.50528 (6)	0.27699 (5)	0.03503 (19)	
O1	0.6980 (2)	0.60686 (17)	0.23887 (13)	0.0318 (5)	
O2	0.9135 (2)	0.63848 (15)	0.36428 (13)	0.0258 (4)	
H2O	0.843 (3)	0.626 (3)	0.3225 (19)	0.054 (13)*	
O3	1.1916 (3)	0.6985 (4)	0.3410 (2)	0.1114 (18)	
H3O	1.2770 (14)	0.706 (5)	0.353 (4)	0.11 (2)*	
N1	0.4760 (2)	0.63643 (17)	0.10133 (15)	0.0232 (5)	
N2	1.1439 (2)	0.64419 (17)	0.49938 (16)	0.0239 (5)	
H2N	1.143 (4)	0.657 (3)	0.4435 (10)	0.040 (10)*	
C1	0.7143 (3)	0.6004 (2)	0.15652 (19)	0.0257 (6)	
C2	0.8383 (3)	0.5781 (2)	0.1389 (2)	0.0345 (7)	
H2	0.9178	0.5676	0.1872	0.041*	
C3	0.8481 (3)	0.5708 (2)	0.0502 (2)	0.0363 (8)	
H3	0.9347	0.5557	0.0401	0.044*	
C4	0.7372 (4)	0.5847 (2)	−0.0217 (2)	0.0344 (7)	
H4	0.7466	0.5793	−0.0810	0.041*	
C5	0.6079 (3)	0.6075 (2)	−0.00683 (19)	0.0280 (6)	
C6	0.5975 (3)	0.6156 (2)	0.08206 (18)	0.0238 (6)	
C7	0.4857 (4)	0.6220 (2)	−0.0757 (2)	0.0324 (7)	
H7	0.4873	0.6172	−0.1366	0.039*	
C8	0.3656 (3)	0.6428 (2)	−0.05527 (19)	0.0303 (7)	
H8	0.2839	0.6530	−0.1020	0.036*	
C9	0.3619 (3)	0.6491 (2)	0.03512 (19)	0.0259 (6)	
C10	0.2318 (3)	0.6695 (2)	0.0599 (2)	0.0322 (7)	
H10A	0.2445	0.7264	0.0992	0.048*	
H10B	0.2074	0.6134	0.0914	0.048*	
H10C	0.1577	0.6823	0.0056	0.048*	
C11	0.8990 (3)	0.61926 (19)	0.44627 (18)	0.0211 (5)	
C12	0.7783 (3)	0.5956 (2)	0.46570 (19)	0.0254 (6)	
H12	0.6953	0.5926	0.4189	0.031*	
C13	0.7759 (3)	0.5758 (2)	0.5544 (2)	0.0267 (6)	
H13	0.6909	0.5594	0.5664	0.032*	
C14	0.8927 (3)	0.5795 (2)	0.62372 (19)	0.0279 (6)	
H14	0.8887	0.5657	0.6831	0.033*	
C15	1.0188 (3)	0.6040 (2)	0.60665 (18)	0.0241 (6)	
C16	1.0214 (3)	0.62310 (19)	0.51737 (18)	0.0218 (6)	
C17	1.1463 (3)	0.6076 (2)	0.6733 (2)	0.0300 (7)	
H17	1.1488	0.5961	0.7342	0.036*	
C18	1.2650 (3)	0.6276 (2)	0.6509 (2)	0.0309 (7)	
H18	1.3496	0.6293	0.6964	0.037*	
C19	1.2646 (3)	0.6457 (2)	0.5617 (2)	0.0284 (6)	
C20	1.3919 (3)	0.6644 (3)	0.5335 (2)	0.0381 (8)	
H20A	1.3932	0.6227	0.4825	0.057*	
H20B	1.4726	0.6504	0.5834	0.057*	
H20C	1.3940	0.7326	0.5158	0.057*	
C21	1.1182 (4)	0.7302 (3)	0.2615 (2)	0.0478 (9)	
H21C	1.1577	0.7911	0.2471	0.072*	
H21B	1.0228	0.7413	0.2632	0.072*	
H21A	1.1200	0.6817	0.2156	0.072*	

### Atomic displacement parameters (Å^2^)

**Table:**

	*U*^11^	*U*^22^	*U*^33^	*U*^12^	*U*^13^	*U*^23^
Zn1	0.02484 (18)	0.0370 (2)	0.01749 (17)	−0.00010 (14)	0.00511 (13)	0.00387 (14)
Cl1	0.0333 (4)	0.0367 (4)	0.0238 (3)	−0.0047 (3)	0.0066 (3)	−0.0003 (3)
Cl2	0.0401 (4)	0.0349 (4)	0.0342 (4)	−0.0009 (3)	0.0170 (3)	0.0076 (3)
O1	0.0251 (11)	0.0528 (14)	0.0170 (10)	−0.0012 (10)	0.0047 (8)	0.0001 (9)
O2	0.0260 (10)	0.0327 (11)	0.0173 (10)	−0.0003 (9)	0.0032 (8)	0.0002 (8)
O3	0.0368 (18)	0.240 (5)	0.063 (2)	0.040 (2)	0.0233 (16)	0.091 (3)
N1	0.0269 (12)	0.0231 (12)	0.0180 (11)	−0.0054 (10)	0.0029 (9)	0.0023 (9)
N2	0.0252 (12)	0.0233 (12)	0.0219 (12)	0.0037 (10)	0.0037 (10)	0.0026 (10)
C1	0.0268 (15)	0.0291 (15)	0.0212 (14)	−0.0070 (12)	0.0063 (11)	−0.0022 (11)
C2	0.0294 (16)	0.0411 (19)	0.0340 (17)	−0.0086 (14)	0.0100 (13)	−0.0070 (14)
C3	0.0348 (18)	0.0404 (19)	0.0404 (19)	−0.0108 (14)	0.0216 (15)	−0.0121 (15)
C4	0.048 (2)	0.0317 (17)	0.0290 (16)	−0.0104 (15)	0.0197 (15)	−0.0057 (13)
C5	0.0413 (17)	0.0217 (14)	0.0222 (14)	−0.0087 (12)	0.0108 (13)	0.0003 (11)
C6	0.0286 (15)	0.0237 (14)	0.0189 (13)	−0.0073 (11)	0.0062 (11)	−0.0001 (10)
C7	0.053 (2)	0.0247 (15)	0.0175 (14)	−0.0061 (14)	0.0055 (13)	−0.0002 (11)
C8	0.0424 (18)	0.0250 (15)	0.0162 (13)	−0.0023 (13)	−0.0050 (12)	0.0019 (11)
C9	0.0306 (15)	0.0206 (14)	0.0230 (14)	−0.0036 (11)	0.0009 (12)	0.0017 (11)
C10	0.0299 (16)	0.0333 (16)	0.0288 (16)	0.0019 (13)	−0.0001 (13)	0.0029 (13)
C11	0.0276 (14)	0.0176 (13)	0.0170 (12)	0.0024 (11)	0.0041 (11)	0.0006 (10)
C12	0.0271 (15)	0.0254 (14)	0.0227 (14)	0.0006 (11)	0.0048 (12)	−0.0022 (11)
C13	0.0295 (15)	0.0253 (15)	0.0281 (15)	0.0009 (12)	0.0123 (12)	0.0011 (12)
C14	0.0379 (17)	0.0252 (15)	0.0210 (14)	0.0042 (13)	0.0086 (12)	0.0017 (11)
C15	0.0306 (15)	0.0196 (13)	0.0209 (14)	0.0044 (11)	0.0049 (11)	−0.0026 (11)
C16	0.0266 (14)	0.0181 (13)	0.0199 (13)	0.0033 (11)	0.0047 (11)	−0.0005 (10)
C17	0.0380 (17)	0.0286 (16)	0.0198 (14)	0.0054 (13)	0.0016 (12)	−0.0013 (12)
C18	0.0282 (15)	0.0321 (16)	0.0260 (15)	0.0031 (13)	−0.0040 (12)	−0.0026 (12)
C19	0.0274 (15)	0.0230 (15)	0.0307 (16)	0.0033 (12)	0.0005 (12)	0.0000 (12)
C20	0.0264 (16)	0.0411 (19)	0.043 (2)	−0.0007 (14)	0.0029 (14)	0.0084 (15)
C21	0.048 (2)	0.057 (2)	0.040 (2)	−0.0010 (18)	0.0137 (17)	0.0023 (18)

### Geometric parameters (Å, °)

**Table:**

Zn1—N1	2.043 (2)	C8—H8	0.9500
Zn1—O1	1.980 (2)	C9—C10	1.488 (4)
Zn1—Cl1	2.2318 (8)	C10—H10A	0.9800
Zn1—Cl2	2.2331 (8)	C10—H10B	0.9800
O1—C1	1.331 (3)	C10—H10C	0.9800
O2—C11	1.342 (3)	C11—C12	1.368 (4)
O2—H2O	0.841 (10)	C11—C16	1.418 (4)
O3—C21	1.329 (5)	C12—C13	1.407 (4)
O3—H3O	0.836 (10)	C12—H12	0.9500
N1—C9	1.332 (4)	C13—C14	1.365 (4)
N1—C6	1.365 (4)	C13—H13	0.9500
N2—C19	1.335 (4)	C14—C15	1.406 (4)
N2—C16	1.367 (4)	C14—H14	0.9500
N2—H2N	0.881 (10)	C15—C16	1.414 (4)
C1—C2	1.382 (4)	C15—C17	1.417 (4)
C1—C6	1.426 (4)	C17—C18	1.359 (5)
C2—C3	1.406 (4)	C17—H17	0.9500
C2—H2	0.9500	C18—C19	1.402 (4)
C3—C4	1.364 (5)	C18—H18	0.9500
C3—H3	0.9500	C19—C20	1.484 (4)
C4—C5	1.417 (5)	C20—H20A	0.9800
C4—H4	0.9500	C20—H20B	0.9800
C5—C7	1.411 (4)	C20—H20C	0.9800
C5—C6	1.412 (4)	C21—H21C	0.9800
C7—C8	1.360 (5)	C21—H21B	0.9800
C7—H7	0.9500	C21—H21A	0.9800
C8—C9	1.413 (4)		
			
O1—Zn1—N1	83.36 (9)	H10A—C10—H10B	109.5
O1—Zn1—Cl1	110.46 (7)	C9—C10—H10C	109.5
N1—Zn1—Cl1	123.24 (7)	H10A—C10—H10C	109.5
O1—Zn1—Cl2	113.75 (7)	H10B—C10—H10C	109.5
N1—Zn1—Cl2	111.22 (7)	O2—C11—C12	125.6 (3)
Cl1—Zn1—Cl2	111.78 (3)	O2—C11—C16	115.8 (2)
C1—O1—Zn1	111.79 (18)	C12—C11—C16	118.5 (3)
C11—O2—H2O	114 (3)	C11—C12—C13	120.6 (3)
C21—O3—H3O	117 (4)	C11—C12—H12	119.7
C9—N1—C6	119.9 (2)	C13—C12—H12	119.7
C9—N1—Zn1	130.4 (2)	C14—C13—C12	121.6 (3)
C6—N1—Zn1	109.52 (18)	C14—C13—H13	119.2
C19—N2—C16	123.6 (3)	C12—C13—H13	119.2
C19—N2—H2N	118 (2)	C13—C14—C15	119.6 (3)
C16—N2—H2N	118 (2)	C13—C14—H14	120.2
O1—C1—C2	123.6 (3)	C15—C14—H14	120.2
O1—C1—C6	118.6 (3)	C14—C15—C16	118.8 (3)
C2—C1—C6	117.8 (3)	C14—C15—C17	124.1 (3)
C1—C2—C3	120.7 (3)	C16—C15—C17	117.0 (3)
C1—C2—H2	119.6	N2—C16—C15	119.5 (3)
C3—C2—H2	119.6	N2—C16—C11	119.7 (2)
C4—C3—C2	122.2 (3)	C15—C16—C11	120.9 (3)
C4—C3—H3	118.9	C18—C17—C15	120.7 (3)
C2—C3—H3	118.9	C18—C17—H17	119.7
C3—C4—C5	119.0 (3)	C15—C17—H17	119.7
C3—C4—H4	120.5	C17—C18—C19	121.0 (3)
C5—C4—H4	120.5	C17—C18—H18	119.5
C7—C5—C6	116.7 (3)	C19—C18—H18	119.5
C7—C5—C4	124.2 (3)	N2—C19—C18	118.1 (3)
C6—C5—C4	119.1 (3)	N2—C19—C20	118.8 (3)
N1—C6—C5	122.2 (3)	C18—C19—C20	123.0 (3)
N1—C6—C1	116.6 (2)	C19—C20—H20A	109.5
C5—C6—C1	121.1 (3)	C19—C20—H20B	109.5
C8—C7—C5	120.3 (3)	H20A—C20—H20B	109.5
C8—C7—H7	119.8	C19—C20—H20C	109.5
C5—C7—H7	119.8	H20A—C20—H20C	109.5
C7—C8—C9	120.2 (3)	H20B—C20—H20C	109.5
C7—C8—H8	119.9	O3—C21—H21C	109.5
C9—C8—H8	119.9	O3—C21—H21B	109.5
N1—C9—C8	120.6 (3)	H21C—C21—H21B	109.5
N1—C9—C10	117.7 (3)	O3—C21—H21A	109.5
C8—C9—C10	121.7 (3)	H21C—C21—H21A	109.5
C9—C10—H10A	109.5	H21B—C21—H21A	109.5
C9—C10—H10B	109.5		
			
N1—Zn1—O1—C1	−2.8 (2)	C5—C7—C8—C9	−0.5 (4)
Cl1—Zn1—O1—C1	−125.81 (18)	C6—N1—C9—C8	−1.0 (4)
Cl2—Zn1—O1—C1	107.55 (19)	Zn1—N1—C9—C8	−175.8 (2)
O1—Zn1—N1—C9	178.1 (3)	C6—N1—C9—C10	178.7 (3)
Cl1—Zn1—N1—C9	−71.7 (3)	Zn1—N1—C9—C10	3.8 (4)
Cl2—Zn1—N1—C9	65.2 (3)	C7—C8—C9—N1	1.0 (4)
O1—Zn1—N1—C6	2.88 (18)	C7—C8—C9—C10	−178.6 (3)
Cl1—Zn1—N1—C6	113.03 (17)	O2—C11—C12—C13	179.2 (3)
Cl2—Zn1—N1—C6	−110.05 (17)	C16—C11—C12—C13	0.1 (4)
Zn1—O1—C1—C2	−176.7 (3)	C11—C12—C13—C14	0.1 (4)
Zn1—O1—C1—C6	2.2 (3)	C12—C13—C14—C15	0.1 (4)
O1—C1—C2—C3	178.9 (3)	C13—C14—C15—C16	−0.7 (4)
C6—C1—C2—C3	0.1 (5)	C13—C14—C15—C17	−178.4 (3)
C1—C2—C3—C4	−0.3 (5)	C19—N2—C16—C15	1.7 (4)
C2—C3—C4—C5	0.1 (5)	C19—N2—C16—C11	−177.4 (3)
C3—C4—C5—C7	−179.0 (3)	C14—C15—C16—N2	−178.1 (3)
C3—C4—C5—C6	0.3 (4)	C17—C15—C16—N2	−0.2 (4)
C9—N1—C6—C5	0.5 (4)	C14—C15—C16—C11	0.9 (4)
Zn1—N1—C6—C5	176.3 (2)	C17—C15—C16—C11	178.9 (2)
C9—N1—C6—C1	−178.4 (3)	O2—C11—C16—N2	−0.8 (4)
Zn1—N1—C6—C1	−2.6 (3)	C12—C11—C16—N2	178.4 (2)
C7—C5—C6—N1	−0.1 (4)	O2—C11—C16—C15	−179.9 (2)
C4—C5—C6—N1	−179.4 (3)	C12—C11—C16—C15	−0.7 (4)
C7—C5—C6—C1	178.8 (3)	C14—C15—C17—C18	177.0 (3)
C4—C5—C6—C1	−0.6 (4)	C16—C15—C17—C18	−0.8 (4)
O1—C1—C6—N1	0.4 (4)	C15—C17—C18—C19	0.5 (5)
C2—C1—C6—N1	179.3 (3)	C16—N2—C19—C18	−2.0 (4)
O1—C1—C6—C5	−178.6 (3)	C16—N2—C19—C20	176.8 (3)
C2—C1—C6—C5	0.4 (4)	C17—C18—C19—N2	0.9 (4)
C6—C5—C7—C8	0.1 (4)	C17—C18—C19—C20	−177.8 (3)
C4—C5—C7—C8	179.4 (3)		

### Hydrogen-bond geometry (Å, °)

**Table:**

*D*—H···*A*	*D*—H	H···*A*	*D*···*A*	*D*—H···*A*
O2—H2O···O1	0.84 (1)	1.70 (1)	2.534 (3)	177 (4)
O3—H3O···Cl1^i^	0.84 (1)	2.47 (3)	3.239 (4)	153 (5)
N2—H2N···O3	0.88 (1)	1.87 (2)	2.727 (4)	163 (3)

Symmetry codes: (i) *x*+1, *y*, *z*.
